# Cell Membrane Integrity in Myotonic Dystrophy Type 1: Implications for Therapy

**DOI:** 10.1371/journal.pone.0121556

**Published:** 2015-03-23

**Authors:** Anchel González-Barriga, Julia Kranzen, Huib J. E. Croes, Suzanne Bijl, Walther J. A. A. van den Broek, Ingeborg D. G. van Kessel, Baziel G. M. van Engelen, Judith C. T. van Deutekom, Bé Wieringa, Susan A. M. Mulders, Derick G. Wansink

**Affiliations:** 1 Department of Cell Biology, Radboud Institute for Molecular Life Sciences, Radboud university medical center, Nijmegen, The Netherlands; 2 Department of Neurology, Donders Centre for Neuroscience, Radboud university medical center, Nijmegen, The Netherlands; 3 Prosensa Therapeutics B.V., Leiden, The Netherlands; University of Valencia, SPAIN

## Abstract

Myotonic Dystrophy type 1 (DM1) is a multisystemic disease caused by toxic RNA from a *DMPK* gene carrying an expanded (CTG•CAG)n repeat. Promising strategies for treatment of DM1 patients are currently being tested. These include antisense oligonucleotides and drugs for elimination of expanded RNA or prevention of aberrant binding to RNP proteins. A significant hurdle for preclinical development along these lines is efficient systemic delivery of compounds across endothelial and target cell membranes. It has been reported that DM1 patients show elevated levels of markers of muscle damage or loss of sarcolemmal integrity in their serum and that splicing of dystrophin, an essential protein for muscle membrane structure, is abnormal. Therefore, we studied cell membrane integrity in DM1 mouse models commonly used for preclinical testing. We found that membranes in skeletal muscle, heart and brain were impermeable to Evans Blue Dye. Creatine kinase levels in serum were similar to those in wild type mice and expression of dystrophin protein was unaffected. Also in patient muscle biopsies cell surface expression of dystrophin was normal and calcium-positive fibers, indicating elevated intracellular calcium levels, were only rarely seen. Combined, our findings indicate that cells in DM1 tissues do not display compromised membrane integrity. Hence, the cell membrane is a barrier that must be overcome in future work towards effective drug delivery in DM1 therapy.

## Introduction

Myotonic Dystrophy type 1 (DM1) is the most common form of muscular dystrophy in adults. Patients with this disease carry an unstable (CTG)n repeat in the 3’ UTR of the *DMPK* gene, the length of which correlates with disease severity [1,2]. DM1’s molecular pathogenesis is complex. Firstly, mutant DMPK RNAs with a long (CUG)n repeat are retained in the cell nucleus, where they abnormally bind transcription and splicing factors, resulting in aberrant protein production and different downstream cellular effects [3]. Secondly, antisense transcripts from the mutant DM1 locus, which carry an expanded (CAG)n repeat, may contribute to the imbalance in proteostasis in DM1 by the production of homopolymeric proteins via a process called RAN translation [4]. Combined, these effects of (CTG•CAG)n expansion are thought to compromise functional development and cause wasting of skeletal muscle (myotonia and muscle weakness), heart (arrhythmia) and brain (mental retardation).

No cure for DM1 is available yet, but strategies for molecular therapy based on antisense RNA, siRNA or oligonucleotides (AONs) [5,6,7,8,9,10,11], compounds that inhibit aberrant (CUG)n RNA-protein interactions [12,13,14] or site-specific RNA endonucleases that target (CUG)n repeats [15] are currently under development. As essentially all these strategies require intracellular delivery of the therapeutic agents (oligonucleotides, high molecular weight organic compounds or proteins), sophisticated approaches may be needed to promote uptake across biological membranes and reach effective tissue concentrations [e.g. 16].

Typically, efficacy of drug uptake into cells is dominantly controlled by molecular characteristics of the cargo itself and by properties of the membranes. Functional changes in cell membranes are central in the pathogenesis of many diseases [17]. For example, membrane permeability can be altered by aberrant protein-membrane interactions, presence of aggregative proteins [18] or lack of integral membrane proteins, i.e. as seen with dystrophin in patients with Duchenne muscular dystrophy (DMD) [19].

Despite its importance as parameter for efficacy of drug delivery, not much is known about membrane integrity in tissues of DM1 patients. Abnormal red blood cell membranes in DM1 patients were noticed in the past [20,21,22]. Pathological features in skeletal muscles include internal nuclei, ring fibers, sarcoplasmic masses, type-I fiber predominance and atrophy, fibrosis and fatty infiltration, and a greatly increased number of intrafusal muscle fibers [23]. Potentially related to the myopathy, DM1 patients may show mildly elevated levels of markers of muscle damage in serum [24], with a possible impact of exercise regimen on these parameters [25]. Finally, aberrant splicing of dystrophin in DM1 patients has been reported [26], with as yet unknown effects on muscle membrane function. All data combined suggest that a certain level of membrane leakiness cannot be excluded in the highly complex DM1 phenotype.

Here, we investigated the possible involvement of membrane permeability in the context of AON-mediated treatment for DM1 using mouse models that replicate DM1 characteristics, i.e. in *HSA* ^LR^ mice [27] and in DM500 [28,29] and DMSXL mice [30], both descendants from the DM300-328 line [31] (Table 1). Better understanding of cell membrane properties in these animal models will support preclinical development of effective therapeutic strategies in DM1 patients. We report on membrane integrity and related membrane characteristics in these models, in comparison to findings in wild type (WT) mice and mdx mice, a DMD mouse model with leaky muscle membranes [32,33]. For comparison, these parameters were also studied in muscle biopsies from DM1 patients. Our study combining mouse and human samples demonstrates that cells in DM1 tissues most likely have a functional membrane. Future therapeutic studies in DM1 mouse models may therefore benefit from advanced targeting strategies for effective therapeutics for DM1.

**Table 1:** Mouse models used in this study.

Mouse model	Transgene/Mutation	Promoter/Expression	Phenotype/Symptoms	Original references
**DM500**	human DM1 locus with ~500 CTG triplets	human *DMPK* promoter/all DM1 related tissues (e.g., skeletal muscle, heart, CNS)	homozygous mice: myopathy, reduced muscle strength, myotonia (generally very mild phenotype)	[28,29]
**DMSXL**	human DM1 locus with ~1600 CTG triplets	human *DMPK* promoter/all DM1 related tissues (e.g., skeletal muscle, heart, CNS)	homozygous mice: myopathy, reduced muscle strength, myotonia, reduced body size (more severe phenotype than DM500 mice)	[30]
***HSA*** ^**LR**^	human *α-actin* gene with ~250 CTG triplets	human *α-actin* promoter/ skeletal muscle only	homozygous mice: strong myotonia, myopathy, no muscle weakness	[27]
**mdx**	spontaneous point mutation in *dystrophin* gene	ubiquitous (e.g., skeletal muscle, heart, CNS, retina)	hemizygous mice: muscle degeneration and atrophy, skeletal muscle fibrosis and necrosis	[32,33]
**WT**	no transgene (genetic background >90% C57BL/6)	n.a.	n.a.	n.a.

## Materials and Methods

### Ethics Statement

Animal experiments were approved by the Animal Ethics Committee of Radboud University (Permit Number: RU-DEC 2012–102).

Human control #1 quadriceps muscle tissue was obtained from the VU University Medical Center (VUmc, Amsterdam, The Netherlands). The use of post-mortem material was approved by the VUmc research committee (project 2011–67), where relatives have given explicit prior written consent that tissue taken at autopsy can be used for research, after completion of the diagnostic process and informed consents were approved by institutional review boards. Human control #2 and #3 quadriceps muscle tissues were obtained from a commercial tissue bank (Cambridge Bioscience, Cambridge, UK; www.bioscience.co.uk). The bank provides frozen human tissue samples that have been collected from pre-consented post-mortem donors in the UK.

Quadriceps muscle biopsies from the six DM1 patients were taken by B. van Engelen in a regular diagnostic procedure, performed routinely in the neuromuscular clinic. Oral informed consent was received from all patients, documented in the patients' charts, to perform the procedure and to use the muscle material for research purposes.

A quadriceps biopsy from a DMD patient was authorized by Dr. M. Tulinius (University of Göteborg, Sweden), with written informed consent for use in research from the patient’s parents, with approval by the Local Ethics Committee in Göteborg.

All biopsies were anonymized before they were handed over for research.

### Human material

All information regarding human muscle samples used in this paper is summarized in Table 2.

**Table 2:** Summary of information on patients and human controls and results from this study.

Sample	Gender	DM1 phenotype	Estimated (CTG)n length in blood	CK value, in U/L (age of sampling)	Age at muscle biopsy, in years	Estimated (CTG)n length in muscle biopsy (see S6 Fig.)	Dystrophin expression in muscle membrane, in a.u. (mean ± CI)	Calcium-positive fibers in muscle biopsy
Control #1	Male	n.a.	n.a.	n.k.	n.k. (adult)	n.a.	146 ± 3	Absent
Control #2	Male	n.a.	n.a.	n.k.	62	n.a.	60 ± 2	Absent
Control #3	Female	n.a.	n.a.	n.k.	83	n.a.	94 ± 2	Rare
DM1 #1	Male	Adult	>200	670 (30), 1182 (32.6), 173 (32.9)	36	~100–300	121 ± 5	Absent
DM1 #2	Male	Adult	n.k.	173 (n.k.)	48	~100–200	103 ± 4	Absent
DM1 #3	Male	Adult (mild)	41–100	735 (n.k.), 1198 (n.k.)	62	~80–400	86 ± 6	Rare
DM1 #4	Male	Adult	>200	n.k.	48	~100–400	146 ± 11	Rare
DM1 #5	Female	Juvenile	n.k.	123 (46)	41	~100–500	n.d.	Absent
DM1 #6	Male	Adult	n.k.	n.k.	48	~80–500	n.d.	Absent
DMD	Male	n.a.	n.a.	n.k.	5	n.a.	7 ± 0.3	Abundant

n.a.: not applicable; n.k.: not known; n.d.: not determined

### Mice

DM500, DMSXL, *HSA* ^LR^ and WT mice (similar background as DM500/DMSXL strains, i.e. >90% C57BL/6) were bred under SPF conditions in the Central Animal Laboratory in Nijmegen. Female homozygous and male hemizygous mdx mice were purchased from The Jackson Laboratory (Bar Harbor, ME, USA). For the DM500, DMSXL and *HSA* ^LR^ lines homozygous mice were used in experiments. From each mouse strain animals of different ages were used (2–6 months for EBD experiments, average 3.5 months; 2–3 months for CK measurements, average 2.2 months) to avoid bias with regard to possible developmental changes. Male and female mice were used to exclude gender-specific effects.

### Exercise regimen in treadmill

A five-lanes treadmill (Panlab/Harvard Apparatus, Holliston, MA, USA) with air-puff control system and 0° slope inclination was used in the exercise regimen (adapted from [34]). Mice were settled with the treadmill belt stationary for 2 minutes, followed by a warm-up period of 8 minutes at a speed of 8 m/minute, immediately followed by the main exercise regimen of 30 minutes at 12 m/minute. Five mice participated in each exercise session (one animal of each strain). All mice performed well in the exercise regimen, except in one session, in which one DMSXL mouse and one mdx mouse stopped running after 20 minutes. In that case the treadmill was stopped and a rest period of 2 minutes was given to all mice. After that, the exercise was continued for 10 minutes without further difficulties.

### Mouse blood collection and serum CK assay

Blood was collected before and after exercise via a tail cut (~100 μl/mouse). Samples were incubated for 1 hour at room temperature to allow clotting and were then centrifuged at 12,000g for 10 minutes at 4°C. Serum was collected and snap frozen in liquid nitrogen before storage at -80°C. Serum samples were diluted 1:10 in 0.9% NaCl prior to analysis. CK activity was determined using the ARCHITECT system (Abbott Laboratories, Abbott Park, IL, USA) based on the IFCC method for the measurement of catalytic activity of CK [35] at the Department of Laboratory Medicine, Radboudumc.

### EBD injection and tissue isolation

Evans Blue Dye (Sigma-Aldrich, St. Louis, MO, USA), 10 μg/μl in physiological saline (0.15 M NaCl, 10 mM phosphate buffer, pH 7.4), sterilized by passage through a 0.2 μm pore size membrane filter was injected intravenously through the tail vein 30 minutes after exercise (50 μl/10 g body weight). Mice were sacrificed by cervical dislocation 24 hours later. Tissues were immediately harvested and frozen in isopentane pre-chilled in liquid nitrogen.

### Tissue cryosectioning

10 μm tissue sections were prepared from a representative middle part of each of the six tissues examined according to standard procedures using a Microm HM 500 Cryostat (Adamas Instrumenten B.V., Rhenen, The Netherlands), dried and stored at -80°C until further use.

### EBD quantification

For EBD imaging, sections were dried at room temperature, dipped in acetone, xylene and mounted in DPX mounting solution (Fisher Scientific, Loughborough, UK). Images were acquired by red autofluorescence in a Leica DMI6000B automated high-content microscope (Leica Microsystems, Wetzlar, Germany) using the TRITC filter. The percentage EBD-positive area was calculated by dividing the EBD-positive area by the total area of the section using ImageJ software.

### Immunohistochemistry

Mouse muscle cryosections were dried at room temperature and incubated for 1 hour in PBS with 0.05% Tween-20 and 5% horse serum, followed by three washing steps in PBS for 5 minutes. Rabbit anti-dystrophin antibody ab15277 (Abcam, Cambridge, UK) was diluted in PBS with 0.05% Tween-20 and 5% fetal bovine serum, applied to the sections and incubated for 2 hours. After washing in PBS 3 times for 5 minutes, sections were incubated with Alexa 488-conjugated goat anti-rabbit in PBS with 0.05% Tween-20 for 1 hour. Samples were rinsed in PBS and then mounted in Mowiol containing 2.5% sodium azide overnight at room temperature. Images were acquired by epifluorescence using the Leica microscope.

Immunostaining and quantification of dystrophin in human samples was done as described [36]. In short, muscle cryosections were dried at room temperature and fixed in acetone for 1 minute, rinsed and washed with PBS for 5 minutes and blocked for 1 hour in PBS with 0.05% Tween-20 and 5% horse serum, followed by another rinsing and washing step in PBS. Rabbit anti-dystrophin ab15277 or IgG isotype antibody (used as control) were diluted in PBS with 0.05% Tween-20 and 5% fetal bovine serum and applied to the sections for 2 hours, followed by 1 hour incubation with ab15277 and mouse anti-spectrin (Novocastra, Newcastle, UK) combined. After rinsing and washing in PBS twice for 5 minutes, sections were incubated with Alexa 488-conjugated goat anti-rabbit and Alexa 594-conjugated goat anti-mouse in PBS with 0.05% Tween-20 for 1 hour. Samples were rinsed and washed twice for 5 minutes in PBS and then mounted using Vectashield (Vector Labs, Burlingame, CA, USA). Images were acquired using the Zeiss LSM 710 confocal microscope (Zeiss, Oberkochen, Germany). The healthy control muscle section displaying the highest dystrophin intensity was used to adjust the confocal settings for the Alexa 488 and 594 channel. These parameters were maintained for all other sections. To minimize variations in laser intensity due to changes in lamp temperature, the laser intensity was kept constant by performing a calibration using a mirror slide. Four to five images per patient or control were acquired, which were subsequently processed using Definiens Architect software (Definiens, Munich, Germany). The software used spectrin signal to locate the membrane of muscle fibers and to define the region of interest. The operator manually excluded areas that were not correctly identified by the program. Dystrophin intensity was calculated in the region of interest, giving a representation of DMD expression per muscle fiber.

### Calcium staining

Muscle sections were dried at room temperature and stained for 5 minutes in 1% Alizarin Red S (Sigma-Aldrich, St. Louis, MO, USA) in distilled water adjusted to pH 5.4 with ammonium hydroxide, as recommended [37]. Samples were dehydrated in acetone (20 dips), incubated in acetone-xylene (1:1) solution (20 dips) and cleared in xylene prior to mounting in DPX mounting solution (Fisher Scientific, Loughborough, UK). Muscle slides were left to dry overnight at room temperature and then stored at 4°C. Images were acquired in the Leica microscope with a TX2 cube and a DFC480 color camera (Leica Microsystems, Wetzlar, Germany).

### (CTG)n length determination by heat pulse extension PCR

Genomic DNA was isolated from muscle sections following standard procedures. To amplify expanded (CTG)n repeats a heat pulse extension PCR protocol was used [38], that allows the generation of DMPK (CTG)n amplicons of up to 1750 CTG repeats. 40 ng DNA was used in a PCR mixture containing 2.25 M betaine (Fluka, Sigma-Aldrich, Germany), 0.2 mM dNTPs (Invitrogen, Carlsbad, CA, USA), 1.33 units DyNAzyme EXT DNA Polymerase (Thermo Scientific, Waltham, MA, USA) and 250 nM of each forward (5´-GCCAGTTCACAACCGCTCCGAGCGTGGGTC-3´) and reverse (5´-ACGCTCCCCAGAGCAGGGCGTCATGC-3´) primers (Biolegio BV, Nijmegen, the Netherlands). Cycling conditions were kept as described [38], except that an annealing temperature of 66°C followed by ramping to 83°C (0.9°C/sec) was used. PCR was performed in a DNA engine Peltier Thermal Cycler (Bio-Rad, Hercules, CA, USA). The size of the expected DMPK amplicon was 324 bp long excluding the (CTG)n repeat.

PCR products were run on a 1% agarose gel and transferred to a Hybond-XL nylon membrane (Amersham, GE Healthcare). The blot was incubated with a ^32^P-end-labeled (CAG)9 probe and after washing exposed to a phosphor imager screen (Kodak, Rochester, NY, USA) for 3 hours. Signal was developed using a Personal FX Phosphor Imager (Bio-Rad).

### Statistics

To compare values of serum CK and EBD-positive fibers between groups, we used a one-way ANOVA test. We applied a paired Student’s t-test to assess whether serum CK activity was significantly different before and after the exercise regimen. All values in graphs are presented as mean ± s.e.m. (unless indicated otherwise). Differences between groups were considered significant when *P*<0.05: *, *P*<0.05; **, *P*<0.01; ***, *P*<0.001. Statistical analyses were performed with GraphPad Prism 5 software (GraphPad Software, Inc., La Jolla, CA, USA).

## Results

For our study of cell membrane integrity we compared WT mice with DM500, DMSXL and *HSA* ^LR^ mice, three transgenic models frequently used in preclinical DM1 studies [39] (Table 1). For an overt DM1 muscle phenotype, all three models were bred to homozygosity. Mdx mice, a well-established model for DMD, were included as positive control, as these demonstrate increased muscle fiber membrane permeability. To minimize possible effects of differences in physical activity between mice and to stimulate muscle contraction, mice were subjected to a 30 minute exercise protocol on a motorized treadmill. Before and after exercise several biomarkers for cell membrane integrity were measured.

### Creatine kinase activity in mouse serum

**Fig 1:**
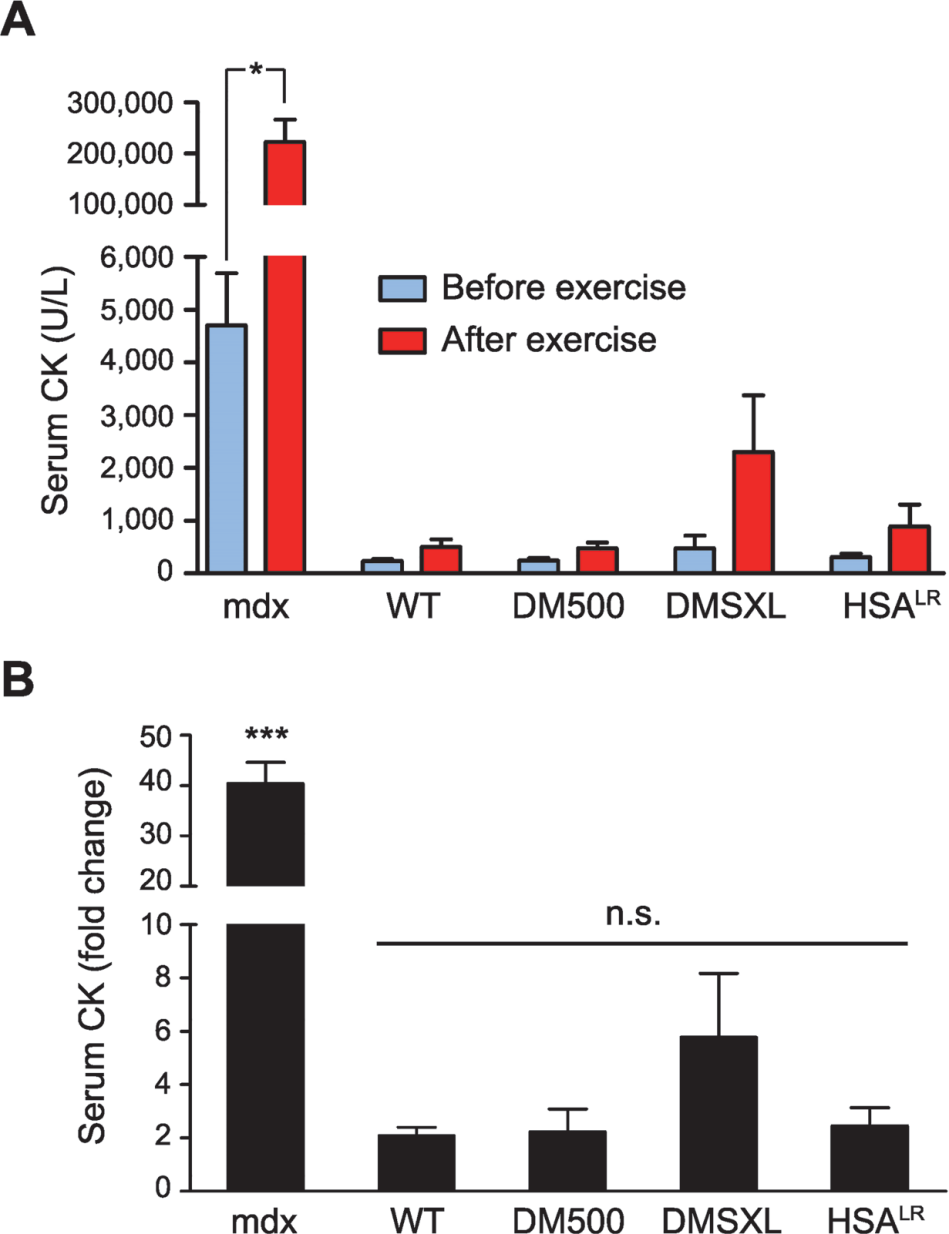
Serum CK level in DM1, mdx and WT mice. (A) CK levels were measured in mouse serum before (blue) and after (red) exercise. CK level was significantly higher in mdx mice than in WT and DM1 model mice, both before and after the exercise regimen (*P*<0.001). Exercise resulted in a significant increase in CK level in mdx mice (*P*<0.05), but not in other mice. (B) Exercise in the treadmill resulted in a forty-fold increase in CK level in mdx mice, whereas only a two-fold increase was observed in WT, DM500 and HSA^LR^ mice and a six-fold increase in DMSXL mice (n = 3–5 per genotype).

Creatine kinase (CK) is released in the circulation upon muscle damage [40] or because of disease-induced loss of membrane integrity. Dystrophin-deficient mdx mice, for example, show an increased basal serum CK level compared to control, which rises significantly after exercise [34]. Under basal conditions, the three DM1 models showed similar serum CK values as WT controls (Fig. 1A). In contrast, the corresponding levels in mdx mice were indeed around 20-fold higher. After exercise, serum CK levels increased only around 2-fold in WT, DM500 and HSA^LR^ animals (Fig. 1B), whereas a 40-fold increase was observed in mdx mice. Markedly enhanced sensitivity of DMSXL mice to exercise was detected, compared to the other DM1 models and WT mice (6-fold increase in CK level; Fig. 1). This effect was probably due to their more severe muscle weakness phenotype, leading to extra muscle wasting necessary to complete the exercise regime, rather than to intrinsic muscle membrane permeability of the model. Nevertheless, neither basal CK levels nor levels after exercise in DMSXL mice were statistically different from those in WT mice.

### Evans Blue Dye uptake in mouse cells *in vivo*

**Fig 2:**
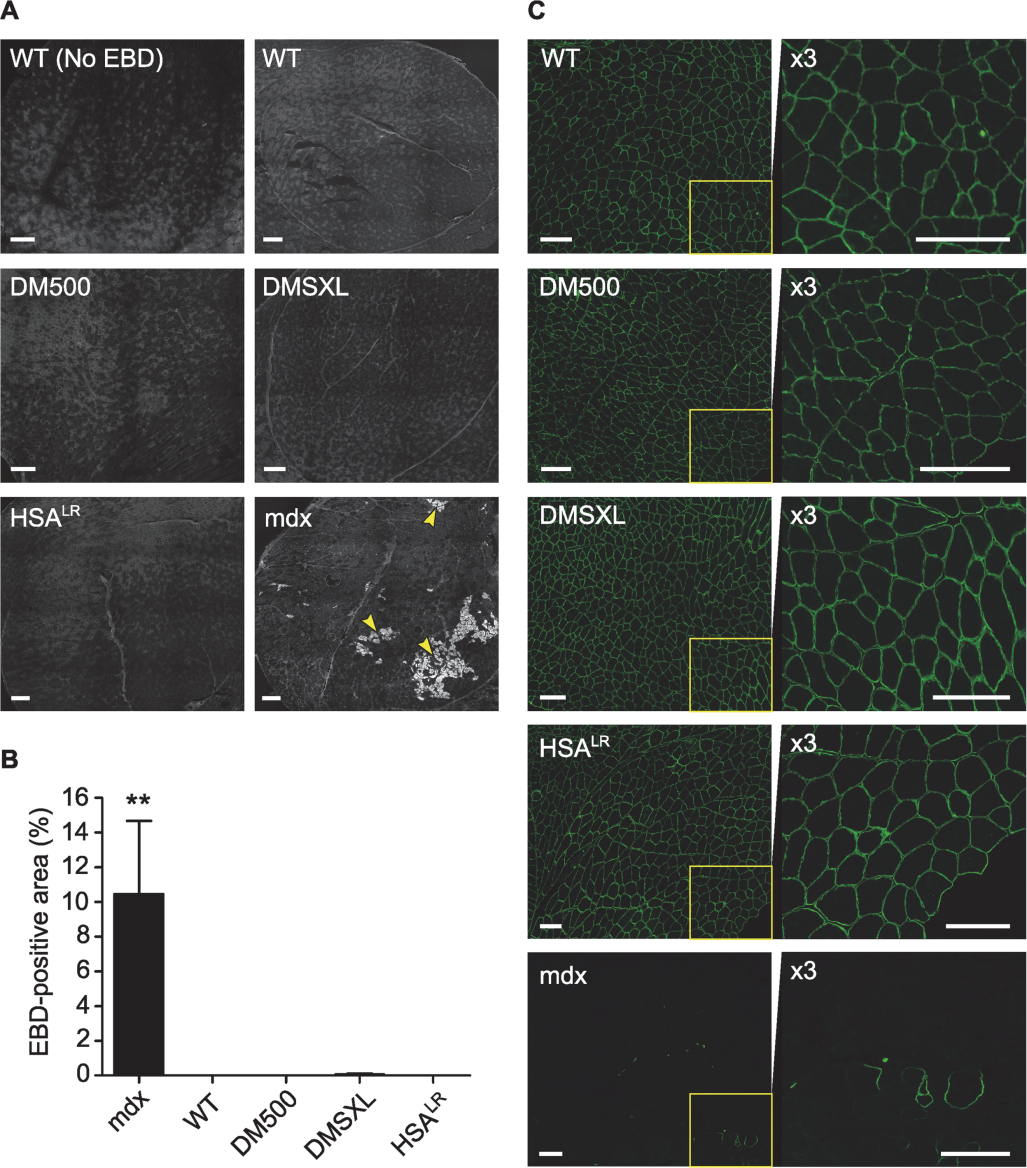
Membrane integrity analysis in mouse quadriceps muscle. (A) Representative images of quadriceps sections from DM1 mice and controls after injection with EBD following exercise. One WT mouse was not injected to appreciate autofluorescent background signal (No EBD). Areas with EBD-positive fibers were regularly seen in mdx samples (arrowheads), but never in WT nor DM1 model samples. Scale bars indicate 250 μm. (B) Quantification of the EBD-positive area compared to total muscle section (n = 4 per group). (C) Representative images of quadriceps sections stained for dystrophin. Staining intensity and pattern observed in WT animals were very similar to those observed in DM500, DMSXL and *HSA* ^LR^ mice. Right panels show high magnifications of insets to appreciate dystrophin staining. As expected, essentially no signal was detected in mdx mice. Scale bars indicate 150 μm.

To evaluate vasculature of living animals and integrity of cell membranes *in vivo* after exercise, we used cell membrane-impermeable Evans Blue Dye (EBD) as tracer [41, 42]. EBD binds to albumin in the bloodstream, leaks into muscle fibers that are damaged and can be observed microscopically by its red autofluorescence. DM1 mice did not show permeability to EBD in any of the skeletal muscles investigated (quadriceps, gastrocnemius, tibialis anterior, diaphragm), similar to WT controls (Fig. 2A,B; S1–S3 Figs.). As expected [41], relatively large, damaged EBD-positive areas were present in mdx muscle. Only once, very few isolated positive fibers were found in a quadriceps muscle of a DMSXL mouse (data not shown). We did not find positive fibers in heart in any of the mice examined (S4 Fig.), although it has been reported that ~50% of mdx mice show EBD-positive fibers in this tissue [41]. Finally, no EBD uptake was observed in brain in any of the mice examined (S5 Fig.).

### Dystrophin expression

Deficiency of dystrophin, a protein involved in muscle membrane structure and flexibility, leads to progressive muscular dystrophy and degeneration in DMD patients and mdx mice [32]. Also in DM1 patients, abnormal production of dystrophin isoforms has been demonstrated [26]. We verified dystrophin protein expression by immunohistochemistry in muscles of exercised DM1 animals and found that the staining pattern was indistinguishable from that in WT mice in all tissues analyzed (Fig. 2C and S1–S4 Figs.). As expected, apart from few so-called revertant fibers [43], no dystrophin staining was detected in mdx mice.

### Cell membrane integrity in DM1 patients

To evaluate our findings obtained in DM1 mouse models, we investigated muscle membrane integrity in human quadriceps muscle. We used biopsies from three healthy controls, six DM1 patients and one DMD patient. (CTG)n repeat lengths in the DM1 muscle biopsies ranged between ~80 and >500, as measured by heat pulse extension PCR [38] (S6 Fig.; Table 2). For some of these patients slightly elevated CK levels had been measured in the past (Table 2).

**Fig 3:**
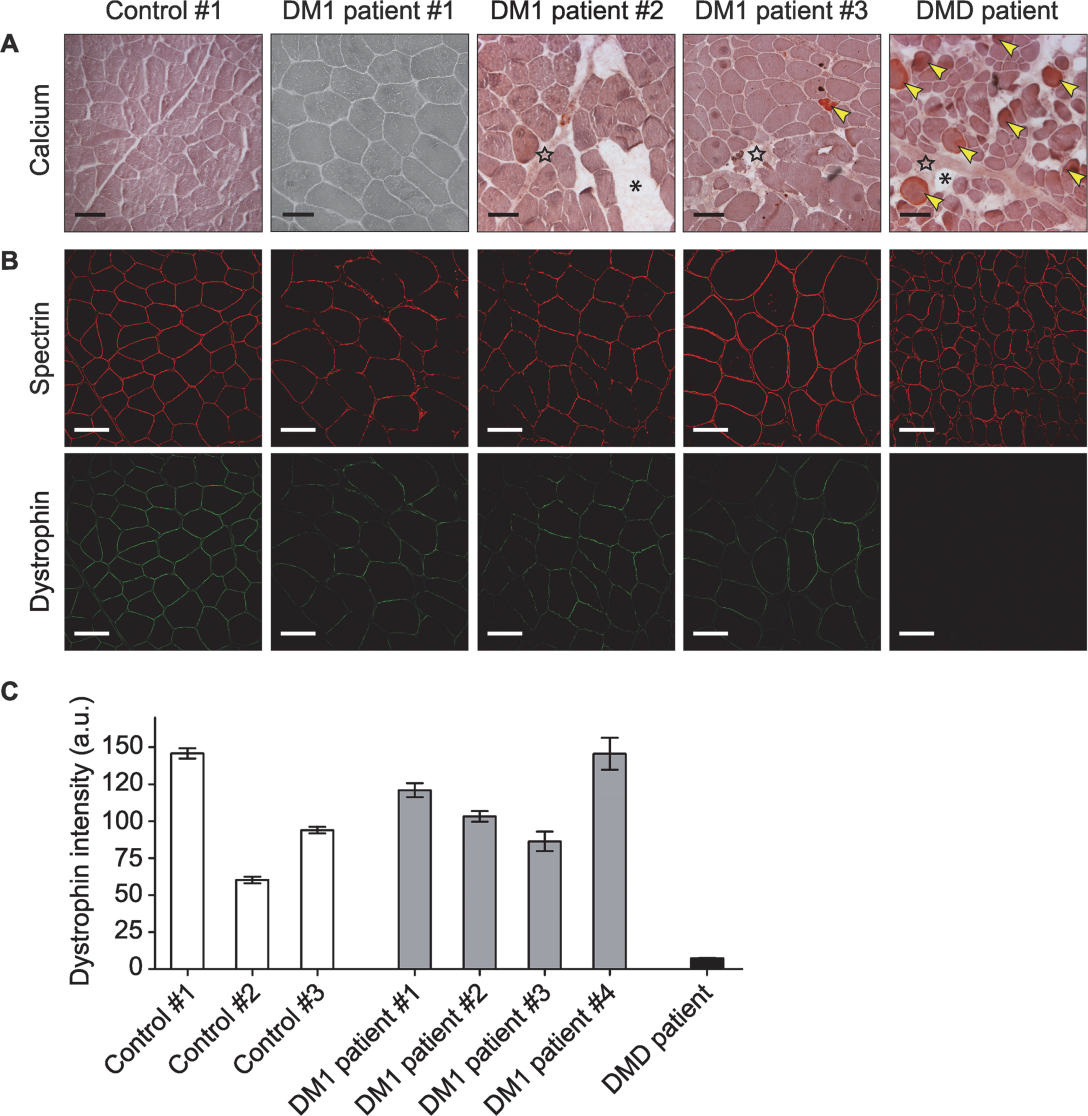
Membrane integrity analysis in DM1 patient biopsies. (A) Calcium staining using Alizarin Red S. No or only few calcium-positive fibers (arrowheads), indicative of abnormal elevated calcium level, were found in DM1 and control biopsies. Calcium deposits were abundant in the DMD sample. DMD muscle sections showed fatty tissue infiltration (asterisks) and fibrosis (stars), which were also detected in some of the DM1 patient biopsies. Scale bars indicate 100 μm. Results from only one of the three controls and three of the six examined DM1 patients are shown. All data are summarized in Table 2. (B) Dystrophin immunostaining. In contrast to the DMD patient biopsy, expression and localization of dystrophin in DM1 and control quadriceps muscles were normal. Spectrin staining was included as a cell membrane reference. Scale bars indicate 100 μm. (C) Quantification of dystrophin expression. Data is represented as mean intensity per fiber (n>50). Error bars indicate 95% confidence interval (CI). Data are summarized in Table 2.

As an alternative method to the EBD injections done in mice, we performed Alizarin Red staining on human muscle sections to detect elevated intracellular calcium levels or deposits, suggestive of membrane abnormalities [37]. Calcium-positive muscle fibers were indeed abundant in DMD muscle (Fig. 3A, Table 2). However, no or only few calcium-positive fibers were observed in controls and DM1 patients.

Next, we performed immunostaining of dystrophin in serial muscle sections from the same patients and controls. Spectrin immunostaining was used to visualize the membrane of muscle fibers. In all healthy control and DM1 biopsies overt cell membrane expression of dystrophin was observed, whereas essentially no dystrophin protein was detected in the DMD sample (Fig. 3B). We quantified the amount of dystrophin using a novel method for immunofluorescence quantification [36]. Dystrophin expression in DM1 samples turned out to be similar to that in control samples (Fig. 3C, Table 2). As expected, dystrophin level in the DMD sample was extremely low.

## Discussion

Efficient systemic cellular uptake of therapeutics is an important determinant for the success of future clinical trials in DM1, because all known molecular targets, e.g., expanded (CUG)n/(CAG)n transcripts or CELF1 and MBNL1 proteins that bind to these repeat-containing RNAs [44] are located intracellularly. The effective concentration of any DM1 drug will therefore depend on its chemical and pharmacokinetic characteristics and the ability to pass plasma membranes of endothelial cells and target cells in muscle, heart or brain of patients. From research on DMD, we have learned that the disease-related loss of dystrophin alters membranes of muscle and brain cells, leading to increased permeability for oligonucleotides and other large molecules [19].

In this study, in comparison to the situation in DMD, we examined cell membrane integrity in DM1 mouse models and in patient muscle biopsies. Our findings on human material suggest that cell membranes in DM1 patients are intact. Expression and localization of dystrophin was normal. We therefore assume that sufficient functionally active protein was present to support membrane function, in spite of an abnormal dystrophin splice mode that affects the protein's c-terminus responsible for interaction with the sarcolemma [26]. Furthermore, in concordance with previous studies [37], calcium deposits, indicative of membrane damage, were rare or absent in DM1 muscle biopsies tested.

In a group of moderately affected, ambulatory DM1 patients one in four male patients and one in two female patients showed mildly elevated basal CK values in serum [24]. Fluctuating, slightly elevated CK levels were also measured in our small cohort of DM1 patients (Table 2). These elevated CK values could be related to a mild membrane defect but, more likely, they are related to myopathy and associated with tissue loss in these patients. More extensive clinical studies in a much larger cohort of individuals are necessary to relate CK values in serum to known variables of DM1 manifestation—e.g. (CTG•CAG)n-repeat length, somatic repeat expansion rate, disease onset and severity, and exercise and life-style habits—before we can draw further conclusions.

The main focus of our study was to obtain better understanding of cell membrane integrity in transgenic DM1 mouse models currently in use for testing new therapies [44]. From a series of observations in models that faithfully replicate most important characteristics of disease in DM1 [39], we conclude that cell membrane function is not affected by presence of repeat RNA. Firstly, we found that serum CK levels in all three models are normal. Even after intense exercise, only a two- to six-fold increase was measured, similar to wild type control animals. Secondly, dystrophin immune-staining was comparable to that in WT mice. Thirdly, we did not observe permeability to EBD in any of the tissues analyzed. With respect to the *HSA* ^LR^ model, our microscopy findings match those presented in an earlier report, where EBD presence was measured in whole muscle lysates by spectrophotometry, using a protocol without exercise regimen [9].

It should be noted that we cannot exclude the possibility that cell membrane integrity in DM1 mice might deteriorate during aging. This aspect was not part of our study and we chose the age group of 2–6 month-old mice, since mice of that age are usually included in therapeutic studies for DM1. For the DMSXL strain in particular, it has been shown that the strongest symptoms are indeed seen in rather young mice [30].

Most of the treatments for DM1 that are currently under development involve the use of relatively large compounds that neutralize expression of expanded DMPK transcripts or block binding to MBNL1 [44, 45]. For these approaches to become effective, active compounds must reach the cell nucleus, i.e., the cellular compartment where most of the toxic (CUG)n RNA is located. Since we find no evidence for membrane alterations or increased membrane permeability in the transgenic DM1 mice, we assume that drugs that were administered systemically and showed efficacy in preclinical studies must have reached their destination via naturally existing cellular uptake and routing mechanisms either independently [9, Mulders et al. unpublished, 12] or promoted by advanced targeting moieties [8, Mulders et al. unpublished]. Based on our findings, we consider it unlikely that the disease state had a major effect on drug fate in these studies, although we do not know whether carrier-mediated transport, receptor-mediated endocytosis or any other transport route was involved [46, 47].

In sum, we conclude that cell membranes of DM500, DMSXL and *HSA* ^LR^ mice are intact and have normal physical stress resistance and properties for chemical passage. Our observations in muscle biopsies from DM1 patients corroborate this picture. Further study is now necessary to know how compounds that have been shown to bind or degrade (CUG)n RNA are internalized by myofibers or other cell types. Future therapeutic studies in DM1 mouse models may benefit from advanced targeting strategies for effective therapeutics for DM1, given the fact that cell membranes are probably intact in patients. As there is no need to concentrate these studies on DM1-affected tissues only, this opens up prospects for the simultaneous development of therapy for other neurodegenerative disorders.

## Supporting Information

S1 Fig Membrane integrity analysis in mouse gastrocnemius muscle. (PDF)

S2 Fig Membrane integrity analysis in mouse tibialis anterior muscle. (PDF)

S3 Fig Membrane integrity analysis in mouse diaphragm muscle. (PDF)

S4 Fig Membrane integrity analysis in mouse heart muscle. (PDF)

S5 Fig Absence of Evans Blue Dye in mouse brain. (PDF)

S6 Fig (CTG)n repeat length determination in human muscle biopsies. (PDF)
