# Supplementary material for: Cell Membrane Integrity in Myotonic Dystrophy Type 1: Implications for Therapy
Source: PLoS One. 2015 Mar 23;10(3):e0121556. doi: 10.1371/journal.pone.0121556 (PMC4370802; doi:10.1371/journal.pone.0121556)
Supplement: S1 Fig — (PDF) [file pone.0121556.s001.pdf]

Supporting Figure S1

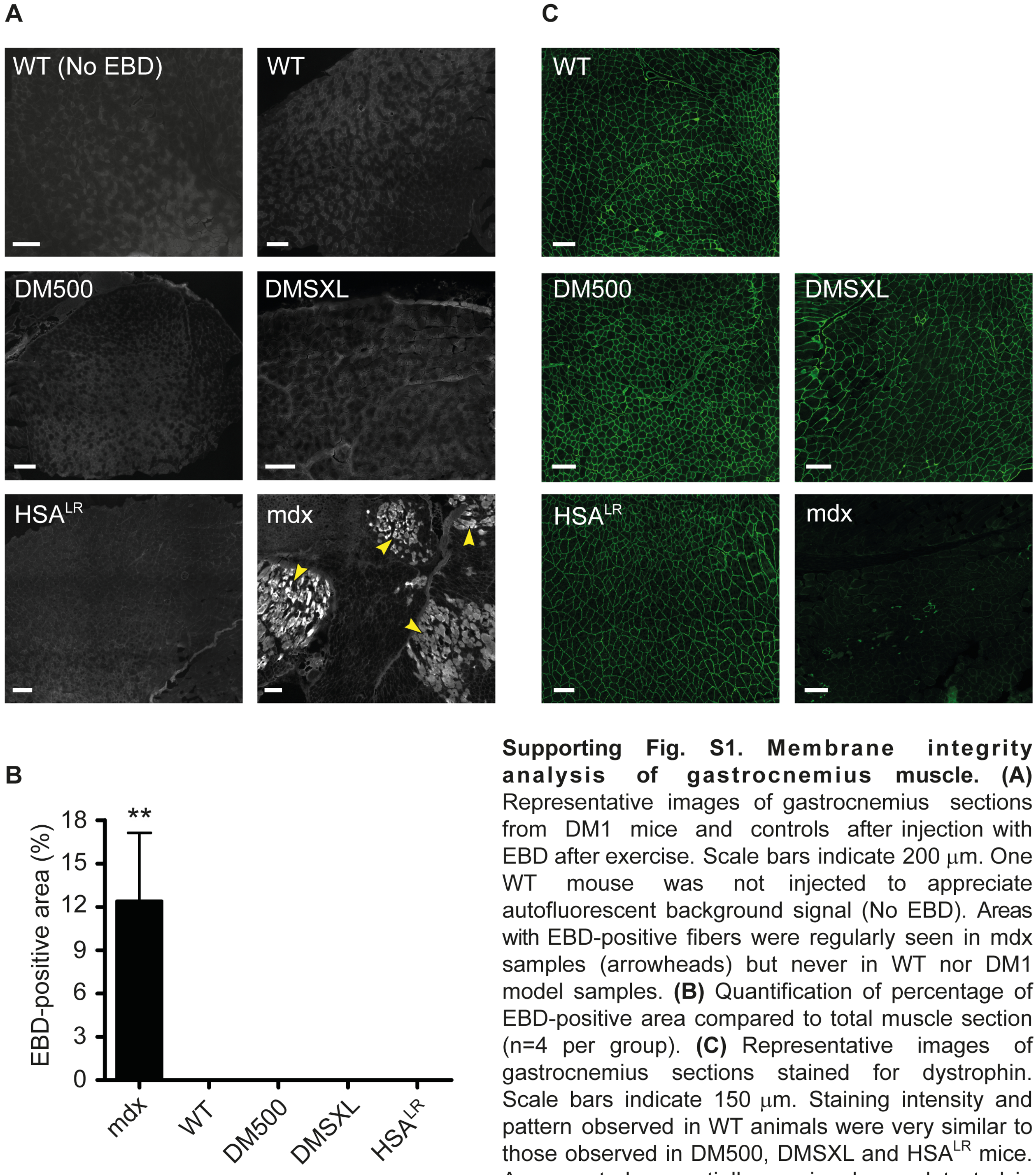

Supporting Fig. S1. Membrane integrity analysis of gastrocnemius muscle. (A) Representative images of gastrocnemius sections from DM1 mice and controls after injection with EBD after exercise. Scale bars indicate 200 μm. One WT mouse was not injected to appreciate autofluorescent background signal (No EBD). Areas with EBD-positive fibers were regularly seen in mdx samples (arrowheads) but never in WT nor DM1 model samples. **(B)** Quantification of percentage of EBD-positive area compared to total muscle section (n=4 per group). **(C)** Representative images of gastrocnemius sections stained for dystrophin. Scale bars indicate 150 μm. Staining intensity and pattern observed in WT animals were very similar to those observed in DM500, DMSXL and HSA^{LR} mice. As expected, essentially no signal was detected in mdx mice.
