# Supplementary material for: Cell Membrane Integrity in Myotonic Dystrophy Type 1: Implications for Therapy
Source: PLoS One. 2015 Mar 23;10(3):e0121556. doi: 10.1371/journal.pone.0121556 (PMC4370802; doi:10.1371/journal.pone.0121556)
Supplement: S4 Fig — (PDF) [file pone.0121556.s004.pdf]

Supporting Figure S4

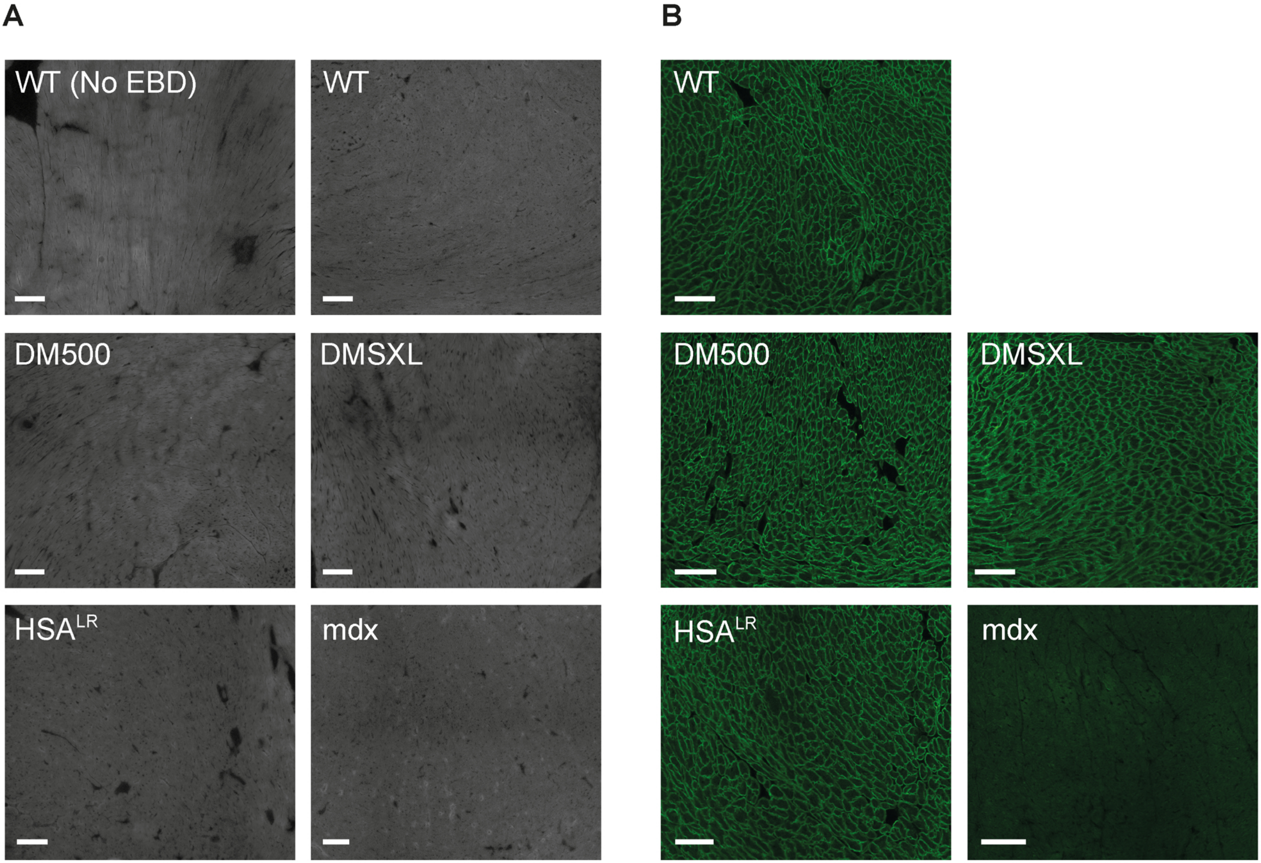

Supporting Fig. S4. Membrane integrity analysis of heart muscle. (A) Representative images of heart sections from DM1 mice and controls after injection with EBD after exercise. Scale bars indicate 100 μm. One WT mouse was not injected to appreciate autofluorescent background signal (No EBD). **(B)** Representative images of heart sections stained for dystrophin. Scale bars indicate 100 μm. Staining intensity and pattern observed in WT animals were very similar to those observed in DM500, DMSXL and HSA^{LR} mice. As expected, essentially no signal was detected in mdx mice.
