# Supplementary material for: Cell Membrane Integrity in Myotonic Dystrophy Type 1: Implications for Therapy
Source: PLoS One. 2015 Mar 23;10(3):e0121556. doi: 10.1371/journal.pone.0121556 (PMC4370802; doi:10.1371/journal.pone.0121556)
Supplement: S5 Fig — (PDF) [file pone.0121556.s005.pdf]

# Supporting Figure S5

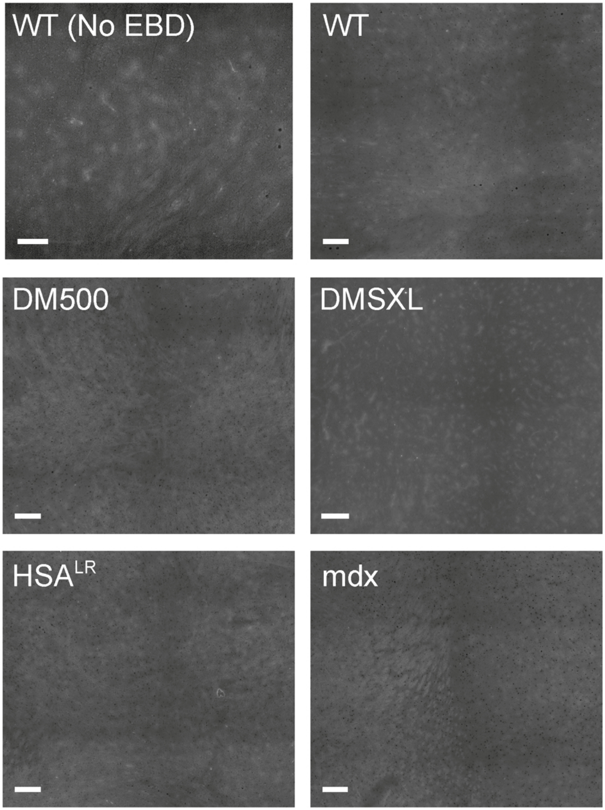


**Supporting Fig. S5. Absence of Evans Blue Dye in brain.** Representative images of mesencephalon (midbrain) from total brain sagittal sections of DM1 mice and controls after injection with EBD after exercise. Scale bars indicate 150 µm. One WT mouse was not injected to appreciate autofluorescent background signal (No EBD). No EBD signal was found in any other brain region either.
