# Supplementary material for: Cell Membrane Integrity in Myotonic Dystrophy Type 1: Implications for Therapy
Source: PLoS One. 2015 Mar 23;10(3):e0121556. doi: 10.1371/journal.pone.0121556 (PMC4370802; doi:10.1371/journal.pone.0121556)
Supplement: S6 Fig — (PDF) [file pone.0121556.s006.pdf]

Supporting Figure S6

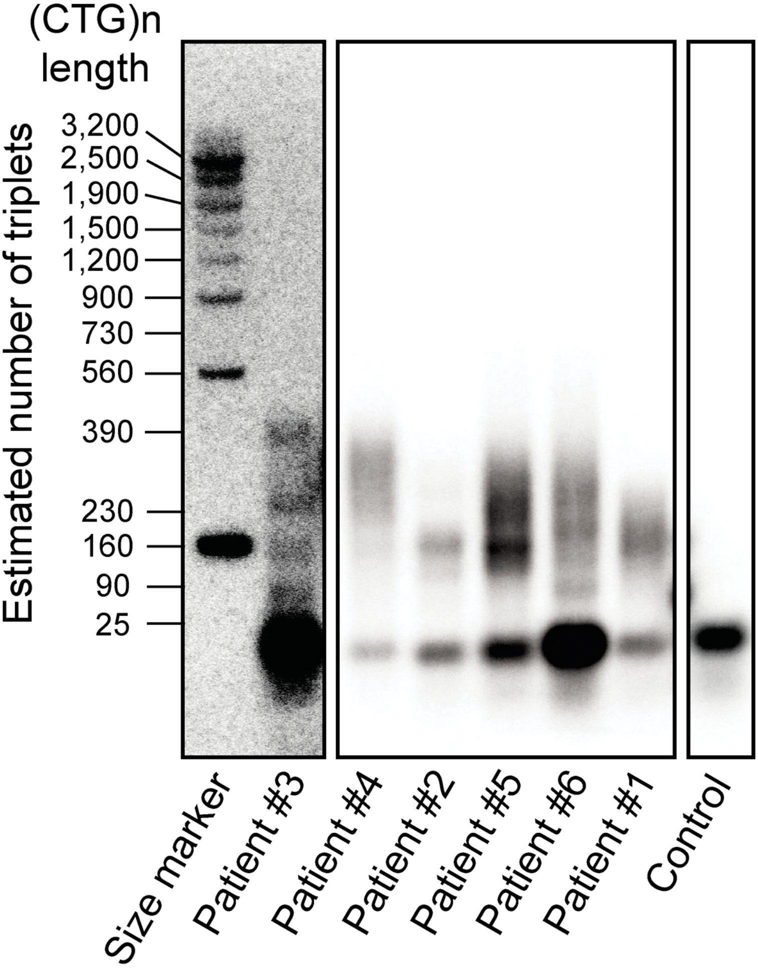

Supporting Fig. S6. (CTG)n repeat length determination in human muscle biopsies. Genomic DNA isolated from human muscle biopsies was used in a heat pulse extension PCR protocol to amplify the (CTG)n repeat of DMPK genes. Control sample represents genomic DNA from a healthy individual carrying a (CTG)5 and a (CTG)12 repeat. The smears in patient samples demonstrate somatic instability. The size marker at the left indicates the estimated number of CTG triplets. Note that lane 1 and 2 were exposed longer for clarity.
